# Mitochondrial Metabolic Reprogramming in Colorectal Cancer-Associated Fibroblasts: An Up-to-Date Review

**DOI:** 10.3390/cancers18111786

**Published:** 2026-05-29

**Authors:** Ying Li, Dipanjan Chanda, Seong-Woo Jeon, Jae-Han Jeon, Min-Ji Kim

**Affiliations:** 1 BK21 Plus KNU Biomedical Convergence Program, Kyungpook National University, Daegu 41944, Republic of Korea; 2 Department of Biomedical Science, Graduate School, Kyungpook National University, Daegu 41944, Republic of Korea; 3 Research Institute of Aging and Metabolism, Kyungpook National University, Daegu 41404, Republic of Korea; 4 Department of Internal Medicine, Kyungpook National University Chilgok Hospital, School of Medicine, Kyungpook National University, 807 Hoguk-ro, Buk-gu, Daegu 41404, Republic of Korea

**Keywords:** colorectal cancer, metabolic reprogramming, tumor microenvironment, cancer-associated fibroblasts, mitochondria

## Abstract

Colorectal cancer (CRC) progression stems from dynamic metabolic crosstalk between malignant cells and the tumor microenvironment (TME). Among stromal components, cancer-associated fibroblasts (CAFs) have emerged as pivotal metabolic drivers rather than mere structural elements. Specifically, evidence indicates that mitochondrial reprogramming in CAFs significantly orchestrates tumor growth, therapeutic resistance, and immune evasion in CRC. This review synthesizes recent insights into how CAF mitochondrial dynamics and metabolic reprogramming dictate CRC biology. We first examine the functional diversity of CAF subpopulations and their distinct mitochondrial requirements. We then contrast mitochondrial dynamics—including fission–fusion balance and mitophagy—between CRC cells and CAFs, highlighting how tumor-derived signals modulate stromal mitochondrial function. We systematically evaluate key regulatory pathways of CAF mitochondrial reprogramming, including TGF-β/HIF-1α, ROS-NF-κB, PI3K–AKT–mTOR, AMPK–PGC-1α, YAP/TAZ mechanotransduction, and mtDNA-mediated cGAS–STING signaling. Furthermore, we discuss how remodeled CAF mitochondria foster metabolic symbiosis via lactate, ketone, and glutamine shuttling; maintain redox homeostasis through the NADPH–glutathione axis and UCP2; and establish immunosuppressive niches via mitochondrial stress signaling. Collectively, these mechanisms drive resistance to chemotherapy, targeted agents, radiotherapy, and immunotherapy. By integrating mitochondrial metabolism, stromal signaling, and clinical responses, this review identifies CAF mitochondria as an actionable target within the CRC TME. Targeting these CAF-specific pathways offers a novel strategy to disrupt tumor–stroma metabolic cooperation and overcome treatment resistance in colorectal cancer.

## Simple Summary

Colorectal cancer (CRC) progression is not only driven by tumor cells themselves but also shaped by their surrounding microenvironment. Among stromal components, cancer-associated fibroblasts (CAFs) play a key role in supporting tumor growth and adaptation. Recent studies suggest that changes in mitochondrial function are closely linked to the metabolic behavior of CAFs. Rather than acting as passive bystanders, CAFs actively undergo metabolic shifts that enable them to provide energy-rich metabolites to tumor cells. In this review, we summarize current knowledge on how mitochondrial alterations in CAFs contribute to metabolic interactions within the tumor microenvironment. We discuss how these changes influence tumor progression, immune regulation, and response to therapy. A better understanding of CAF metabolism, particularly at the mitochondrial level, may offer new opportunities for developing more effective treatment strategies in CRC.

## 1. Introduction

As one of the most prevalent malignancies, colorectal cancer (CRC) is responsible for the second highest number of cancer-related deaths worldwide [1,2]. However, conventional treatments have not significantly reduced the mortality rate [3,4]. However, the emergence of drug resistance substantially limits the clinical efficacy of current therapeutic strategies for colorectal cancer [5], highlighting the urgent need to identify novel therapeutic targets and improve precision treatment approaches.

Mitochondria play complex and crucial roles in the tumor microenvironment (TME) of CRC, and their functions go far beyond the traditional concept of being an “energy factory”. In addition to ATP production, mitochondria participate in multiple cellular processes, including apoptosis regulation, oxidative stress control, and inflammatory signaling, all of which are closely linked to the development and progression of CRC [6]. Within the TME, mitochondrial alterations influence not only tumor cells but also surrounding stromal components, including cancer-associated fibroblasts (CAFs) and immune cells. For instance, the metabolic reprogramming of mitochondria in CAF can enhance their tumor-promoting properties, including the secretion of growth factors and remodeling of the extracellular matrix [7]. Therefore, in-depth investigation of the roles of mitochondria in the TME of CRC, as well as exploration of therapeutic strategies targeting mitochondrial metabolism or function, is of great significance for improving CRC treatment outcomes. Glycolysis and oxidative phosphorylation (OXPHOS) are the two main sources of energy that cancer cells use for growth and metastasis. Evidence indicates that tumor expansion and malignancy are contingent upon mitochondrial function [8], a fundamental driver of metabolic flexibility in neoplastic cells. Even in the presence of sufficient oxygen, neoplastic cells frequently undergo a metabolic transition known as the Warburg effect, which is a hallmark metabolic feature of cancer cells, resulting in enhanced glucose uptake and lactate production. Although this metabolic shift is less efficient for ATP generation, it supports rapid proliferation by providing intermediates for biosynthetic pathways and promoting adaptation to the tumor microenvironment, prioritizing aerobic glycolysis over the more energy-efficient oxidative phosphorylation pathway [9]. This suggests that cancer growth and progression depend on mitochondrial activity. While glycolysis has long been emphasized as a dominant metabolic feature of cancer cells, mitochondria remain central regulators of cellular energy balance, biosynthetic capacity, and redox signaling. Consistent with this notion, cancer-associated mitochondrial alterations—various mitochondrial alterations have been reported, including changes in membrane potential, mitochondrial dynamics, and reactive oxygen species production [10]. Meanwhile, increasing interest has emerged in the role of stromal components within the TME, particularly CAFs, which represent one of the most abundant and functionally versatile non-malignant cell populations in CRC. CAFs actively remodel the extracellular matrix, modulate metabolic networks, suppress antitumor immune responses, and maintain tumor cell stemness, thereby promoting oncogenic proliferation, metastasis and resistance to therapy [11].

**Figure 1:**
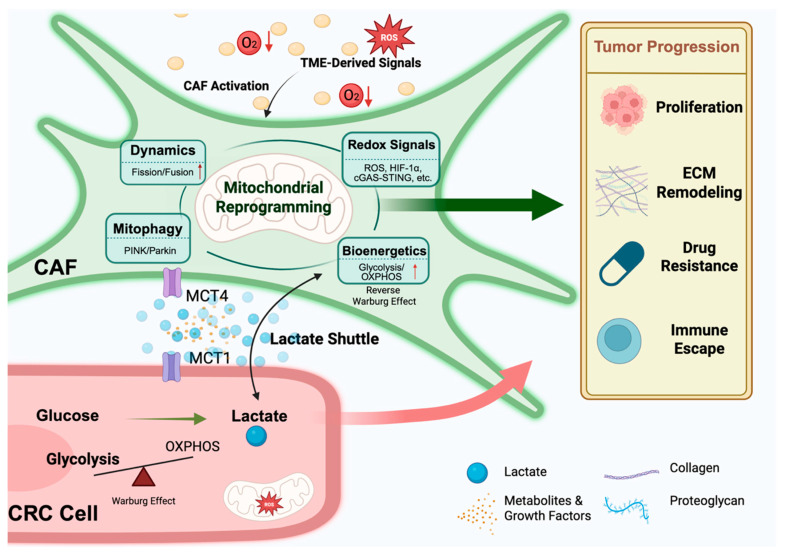
CAF Mitochondrial Reprogramming Drives Tumor Progression. Cancer-associated fibroblasts (CAFs) are activated by tumor microenvironment (TME)-derived signals, including cytokines, reactive oxygen species (ROS) and hypoxia. In colorectal cancer (CRC), tumor cells predominantly rely on aerobic glycolysis (Warburg effect), leading to increased lactate production. Lactate is transported between CRC cells and CAFs via monocarboxylate transporters (MCT1 and MCT4), establishing a metabolic coupling that supports CAF bioenergetic remodeling and contributes to the reverse Warburg effect. Within CAFs, mitochondrial reprogramming acts as a central regulatory hub integrating multiple processes, including mitochondrial dynamics, mitophagy, bioenergetic adaptation and redox signaling pathways. The red upward arrows indicate enhanced mitochondrial activity and metabolic adaptation, including increased glycolytic flux and altered fission–fusion dynamics within CAFs. These coordinated mitochondrial alterations promote the production and secretion of metabolites, growth factors, and extracellular matrix (ECM) components. Functionally, CAF mitochondrial reprogramming may contribute to tumor progression by enhancing cancer cell proliferation, ECM remodeling, drug resistance, and immune evasion. Collectively, these findings highlight CAF mitochondria as key mediators of tumor–stroma interactions and potential targets for therapeutic intervention. Created in BioRender. Jeon, J. (2026) https://BioRender.com/94jartm (accessd on 4 May 2026). Abbreviations: CAF, cancer-associated fibroblast; CRC, colorectal cancer; TME, tumor microenvironment; mtDNA, mitochondrial DNA; ROS, reactive oxygen species; HIF-1α, hypoxia-inducible factor 1-alpha; NF-κB, nuclear factor kappa B; cGAS, cyclic GMP–AMP synthase; STING, stimulator of interferon genes; OXPHOS, oxidative phosphorylation; MCT1, monocarboxylate transporter 1; MCT4, monocarboxylate transporter 4.

In contrast, despite increasing recognition of metabolic reprogramming in tumor, the specific role of mitochondrial remodeling in colorectal CAFs remains poorly defined. How tumor-driven alterations in CAFs mitochondrial metabolism shape metabolic symbiosis and stromal–tumor crosstalk—and ultimately influence therapeutic responses—has not been systematically addressed. In this article, we focused on the emerging role of CAF-associated mitochondrial remodeling in shaping metabolic adaptation, stromal heterogeneity, therapeutic resistance, and tumor progression within the CRC microenvironment, while also discussing the translational potential of targeting CAF mitochondrial signaling pathways, highlighting how mitochondrial remodeling in CAFs contributes to tumor–stromal metabolic interactions and therapy resistance in CRC (Figure 1). Importantly, we distinguish between mechanisms directly validated in colorectal CAFs and those inferred from stromal biology in other tumor types, emphasizing areas where CRC-specific evidence remains limited and requires further investigation.

## 2. Mitochondrial Dynamics in Colorectal Cancer Cells and CAFs

### 2.1. Mitochondrial Dynamics in CRC Tumor Cells

Mitochondria are dynamic organelles that continually undergo fusion and fission to fulfill their roles and respond to cellular needs. In CRC, growing evidence indicates that these dynamic characteristics have been significantly altered, resulting in increased mitochondrial division and fragmentation [12]. This fragmentation, characterized by a shift in the balance between fission and fusion, leads to a disrupted mitochondrial network. This morphological shift is not only a hallmark of the metabolic switch but also actively promotes enhanced glycolysis and ROS production by diminishing the efficiency of oxidative phosphorylation [13,14]. While mitochondrial fusion proteins are often suppressed in aggressive CRC clones, the overactivation of fission machinery remains the dominant feature of the TME. Specifically, during cancer cell proliferation, the fission mediated by dynamin-related protein 1 (DRP1) facilitates the division of the mitochondrial network, ensuring that daughter cells inherit mitochondria equally and promoting continuous growth [15]. Tumor-derived TGF-β and other factors can promote DRP1 phosphorylation at Ser616, thereby triggering excessive mitochondrial fission [16].

Further evidence may suggest that the heightened activity of DRP1 in CRC contributes to metabolic reprogramming. The increased fragmentation of mitochondria in CRC promotes the glycolytic pathway, supports fatty acid oxidation (FAO) signaling, and regulates the secretion of tumor-promoting factors [17,18]. In addition to malignant cells, mitochondrial remodeling has also been observed in stromal fibroblasts exposed to tumor-derived oxidative stress. Increased mitophagy and metabolic conversion of fibroblasts toward aerobic glycolysis have been documented, suggesting that mitochondrial homeostasis is profoundly altered in these stromal cells. Although direct evidence specifically addressing fission–fusion imbalance in CRC-associated fibroblasts remains limited, these observations indicate that mitochondrial dynamics may contribute to stromal metabolic adaptation [19]. Collectively, these findings suggest that mitochondrial fragmentation supports metabolic flexibility in CRC cells and may also influence stromal adaptation within the TME. Given the central role of CAFs in metabolic coupling and tumor progression, clarifying how mitochondrial dynamics regulate fibroblast function represents an important area for future investigation.

### 2.2. Interaction of Mitochondrial Dynamics Between CRC Cells and CAFs

**Figure 2:**
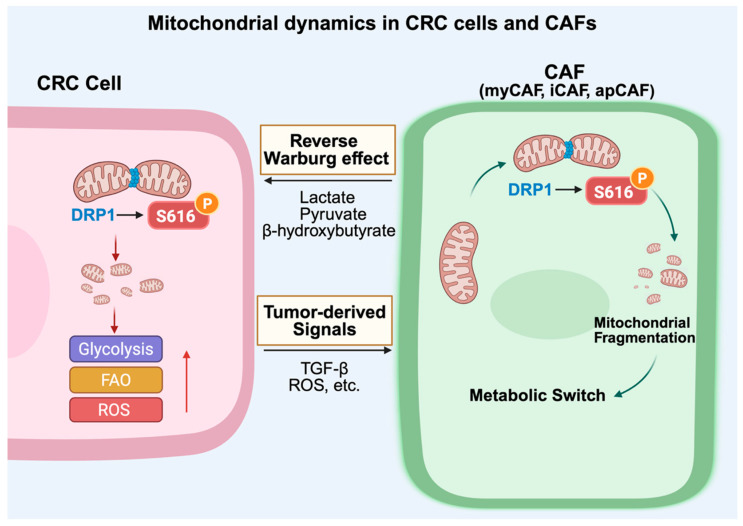
Schematic model of mitochondrial dynamics–mediated metabolic coupling between CRC cells and CAFs. Schematic illustration showing the interaction between colorectal cancer (CRC) cells and cancer-associated fibroblasts (CAFs) through mitochondrial dynamics in the tumor microenvironment. In CRC cells, increased DRP1-mediated mitochondrial fission promotes mitochondrial fragmentation and metabolic reprogramming, leading to enhanced glycolysis, fatty acid oxidation (FAO), and reactive oxygen species (ROS) production. CRC cells release tumor-derived signals, including transforming growth factor-β (TGF-β), ROS, and other cytokines, which stimulate DRP1 phosphorylation (Ser616) and mitochondrial fragmentation in CAFs. This remodeling shifts CAF metabolism from oxidative phosphorylation (OXPHOS) toward aerobic glycolysis, generating metabolites such as lactate, pyruvate, and β-hydroxybutyrate. These metabolites are transferred to CRC cells to support the TCA cycle and OXPHOS, thereby promoting tumor growth. CAFs exhibit functional heterogeneity, including myofibroblastic (myCAF), inflammatory (iCAF), and antigen-presenting (apCAF) subtypes. Created in BioRender. Jeon, J. (2026) https://BioRender.com/94jartm (accessd on 4 May 2026). Abbreviations: CRC, colorectal cancer; CAF, cancer-associated fibroblast; myCAF, myofibroblastic cancer-associated fibroblast; iCAF, inflammatory cancer-associated fibroblast; apCAF, antigen-presenting cancer-associated fibroblast; DRP1, dynamin-related protein 1; p-DRP1 (Ser616), phosphorylated DRP1 at serine 616; TGF-β, transforming growth factor-beta; ROS, reactive oxygen species; FAO, fatty acid oxidation.

Both CRC cells and CAFs can undergo mitochondrial division. Recent single-cell transcriptomic analyses have further highlighted the substantial heterogeneity of CAF populations within the CRC tumor microenvironment, identifying functionally distinct subtypes including myofibroblastic CAFs (myCAFs), inflammatory CAFs (iCAFs), and antigen-presenting CAFs (apCAFs) [20]. MyCAFs are primarily associated with extracellular matrix remodeling, contractile activity, and tissue stiffening, processes that likely require sustained mitochondrial ATP production and active oxidative metabolism. In contrast, iCAFs exhibit enhanced secretion of inflammatory cytokines such as IL-6 and CXCL12 and may preferentially adopt glycolytic metabolic programs to support their secretory phenotype. Moreover, recent evidence suggests that CAF phenotypes in CRC are dynamically regulated by signaling pathways such as Wnt, which can induce phenotypic switching and functional plasticity among stromal populations [21]. Although the metabolic characteristics of apCAFs remain incompletely understood, their immunomodulatory properties suggest potential links to mitochondrial stress signaling and antigen-processing pathways. Collectively, these findings indicate that metabolic and mitochondrial heterogeneity may represent an additional layer of functional specialization among CAF subtypes in CRC. However, direct comparisons of mitochondrial dynamics and metabolic flux across CAF subpopulations remain limited and warrant further investigation. And how mitochondrial dynamics differ among these CAF subtypes remains poorly understood. Instead, evidence suggests that signals from CRC cells can influence the mitochondrial state of adjacent CAFs [22]. This interplay serves as a pivotal driver of intercellular signaling and coordination throughout the TME. CRC cells are known to release various soluble factors, including TGF-β and reactive oxygen species (ROS), which have been shown to affect fibroblasts and promote mitochondrial fragmentation [23,24]. When CAFs receive these signals, they activate proteins involved in mitochondrial fission, such as DRP1, which becomes phosphorylated at the serine 616 site. This modification facilitates DRP1’s translocation to the outer mitochondrial membrane, enhancing mitochondrial division in fibroblasts [25]. As mitochondrial fragmentation becomes more pronounced, CAFs typically transition from oxidative phosphorylation (OXPHOS) to aerobic glycolysis, a metabolic pattern referred to as the Reverse Warburg Effect [26,27]. With the increase in glycolysis, CAFs begin to produce more metabolites, including lactate, pyruvate, and certain ketone bodies (e.g., β-hydroxybutyrate). These metabolites can be utilized by nearby CRC cells to support the TCA cycle and OXPHOS, enabling cancer cells to more effectively satisfy the metabolic and biosynthetic requirements essential for proliferation (Figure 2).

## 3. Signaling Pathways Regulating Mitochondrial Reprogramming in CRC-Associated Fibroblasts

**Figure 3:**
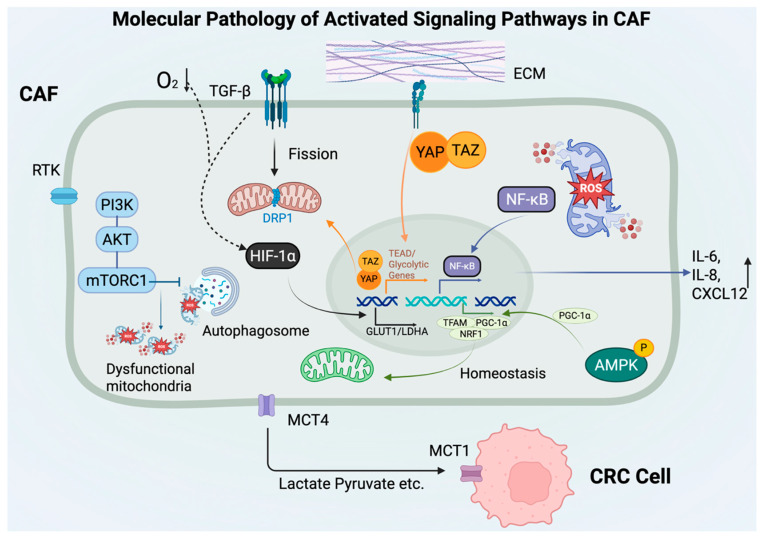
Mitochondria-associated signaling pathways regulating metabolic reprogramming in cancer-associated fibroblasts in colorectal cancer. This schematic summarizes representative signaling networks activated in cancer-associated fibroblasts (CAFs) in response to tumor microenvironment (TME)–associated stresses. TGF-β/HIF-1α axis: Hypoxia and TGF-β signaling are associated with DRP1-mediated mitochondrial fission and HIF-1α stabilization, which facilitating the transcriptional activation of key glycolytic effectors, including the glucose transporter GLUT1 and the enzyme LDHA. YAP/TAZ pathway: CAFs sense extracellular matrix (ECM) stiffness through integrin signaling, leading to YAP/TAZ nuclear translocation and TEAD-dependent transcriptional programs linked to glycolytic regulation. ROS/NF-κB pathway: Mitochondrial dysfunction can increase reactive oxygen species (ROS) levels, which are associated with NF-κB activation and the expression of pro-inflammatory cytokines, including IL-6, IL-8, and CXCL12. AMPK/PGC-1α pathway: This pathway functions as a metabolic rheostat that supports mitochondrial biogenesis and cellular homeostasis under metabolic stress. PI3K/AKT/mTOR axis: The constitutive activation of mTORC1 correlates with suppressed mitophagic flux, leading to the intracellular buildup of compromised mitochondria. Metabolic symbiosis: CAFs facilitate the efflux of lactate and pyruvate through MCT4, which are subsequently internalized by CRC cells via MCT1 to fuel mitochondrial oxidative phosphorylation (OXPHOS). Created in BioRender. Jeon, J. (2026) https://BioRender.com/3yxj06a (accessd on 4 May 2026). Abbreviations: CAF, cancer-associated fibroblast; CRC, colorectal cancer; ECM, extracellular matrix; RTK, receptor tyrosine kinase; PI3K, phosphoinositide 3-kinase; AKT, protein kinase B; mTORC1, mechanistic target of rapamycin complex 1; AMPK, AMP-activated protein kinase; HIF-1α, hypoxia-inducible factor 1-alpha; TGF-β, transforming growth factor-beta; DRP1, dynamin-related protein 1; YAP, Yes-associated protein; TAZ, transcriptional co-activator with PDZ-binding motif; TEAD, TEA domain transcription factor; NF-κB, nuclear factor kappa B; ROS, reactive oxygen species; GLUT1, glucose transporter 1; LDHA, lactate dehydrogenase A; TFAM, mitochondrial transcription factor A; NRF1, nuclear respiratory factor 1; PGC-1α, peroxisome proliferator-activated receptor gamma coactivator 1-alpha; MCT1, monocarboxylate transporter 1; MCT4, monocarboxylate transporter 4; IL-6, interleukin-6; IL-8, interleukin-8; CXCL12, C-X-C motif chemokine ligand 12.

Many of the signaling pathways discussed below have been increasingly recognized as conserved regulators of stromal metabolic adaptation across multiple solid tumors. While the extent of direct validation in CRC-associated fibroblasts varies among pathways, accumulating evidence supports the relevance of these mitochondrial and metabolic programs within the colorectal microenvironment. In CRC, the metabolic activity of CAFs plays a crucial role in tumor progression. This metabolic shift, particularly within the mitochondria, is not random; it is driven by a network of signaling pathways [28,29]. These pathways are activated by various cues from the tumor, including secreted factors and conditions of metabolic stress. Our understanding of these mechanisms is informed by both direct evidence from CAFs in CRC and insights drawn from CAF biology in other cancer types. The functional outcomes are significant: these signals reshape mitochondrial structure and function, alter energy production pathways, disrupt redox balance, and increase the secretion of metabolites that promote tumor growth [8]. In this summary, we outline the principal pathways believed to govern mitochondrial remodeling in the CAFs of CRC (Figure 3).

### 3.1. TGF-β/HIF-1α Axis

The cytokine TGF-β is a major driver of CAF activation in the CRC microenvironment. Among its many effects, TGF-β signaling induces significant changes in mitochondrial dynamics. It promotes mitochondrial fission—a process mediated by increased DRP1 activity—while simultaneously reducing the fusion proteins’ expression like Mitofusin-1 (MFN1) and Mitofusin-2 (MFN2) [30,31,32]. Consequently, mitochondrial fission correlates with elevated reactive oxygen species (ROS) generation and a metabolic transition that prioritizes glycolytic flux over oxidative phosphorylation. This shift is further reinforced by Hypoxia-Inducible Factor-1 Alpha (HIF-1α). In the hypoxic and nutrient-poor niches of CRC tumors, HIF-1α is stabilized and acts as a master regulator of glycolysis. It transcriptionally upregulates LDHA, PDK1, and GLUT1 [33,34], effectively enhancing glycolytic flux and limiting mitochondrial pyruvate utilization. Beyond its cell-intrinsic metabolic effects, HIF-1α also promotes the expression of VEGFA and matrix-remodeling enzymes like MMP9 [35], thereby linking CAF metabolism to broader processes of angiogenesis and stromal remodeling. Evidence from CRC studies indicates that the TGF-β/HIF-1α axis contributes to metabolic reprogramming in both tumor cells and CAFs [36,37].

### 3.2. ROS/NF-κB Signaling

Increased reactive oxygen species (ROS) generation is commonly observed in activated CAFs in CRC. These ROS can originate from several sources, including a fragmented mitochondrial network, electron transport chain leakage as well as the activity of ROS-producing enzymes such as NADPH oxidase 4 (NOX4) [38]. This redox imbalance serves as a signaling mechanism; moderate ROS levels can activate the NF-κB transcription factor. Once activated, NF-κB drives CAFs to produce and secrete a range of cytokines, including IL-6, IL-8, and CXCL12, which promote cancer cell proliferation, stem-like properties, and invasion [37,39]. Additionally, NF-κB activation modifies the metabolic interactions between CAFs and tumor cells, increasing the export of energy substrates like lactate and pyruvate [40]. This creates a self-reinforcing cycle: persistent ROS production maintains mitochondrial stress and a glycolytic state in CAFs, which, in turn, supports tumor progression through both signaling and metabolic pathways, consistent with this metabolic adaptation model, mitochondrial dysfunction-associated stress has also been implicated in promoting glycolytic reprogramming in CAFs. Notably, mitochondrial fission was reported to induce glycolytic activation and stromal lactate production in cancer-associated myofibroblasts, thereby supporting early tumor growth [19]. Although direct evidence in CRC-associated fibroblasts remains limited, these findings further support the potential link between mitochondrial stress, metabolic adaptation, and tumor-promoting stromal crosstalk. Observations in CRC models are consistent with this mechanism.

### 3.3. mtDNA/cGAS-STING Pathway

Mitochondrial distress within CAFs can induce the translocation of mitochondrial DNA (mtDNA) into the cytoplasmic compartment. The liberation of mitochondrial DNA into the cytoplasm triggers recognition by the cGAS sensor, subsequently activating the STING-dependent signaling axis to stimulate the production of type I interferons and a repertoire of pro-inflammatory cytokines [13]. mtDNA released into the extracellular environment can be internalized by immune cells and recognized by Toll-like receptors, including TLR9. This recognition triggers the secretion of immunosuppressive cytokines like IL-6 and IL-10 [41,42]. Although the complete sequence of these events has not been thoroughly studied in CRC CAFs specifically, existing literature on stromal biology in other cancers supports this pathway to a certain extent. Although mtDNA-mediated cGAS–STING activation has been implicated in stromal inflammation and immune modulation in several malignancies, direct evidence demonstrating this signaling cascade specifically in CRC-associated fibroblasts is currently lacking. Therefore, its contribution to CRC stromal immunosuppression should presently be considered hypothetical and warrants further experimental validation.

### 3.4. AMPK–PGC-1α Energy-Sensing Pathway

AMPK serves as a fundamental molecular rheostat, monitoring and responding to fluctuations in the cellular energy state. In fibroblasts within the stressful TME, AMPK is activated in response to low nutrient availability or high oxidative stress. Upon activation, AMPK stimulates mitochondrial biogenesis by upregulating PGC-1α, a master regulator that modulates the expression of mitochondrial-encoded genes by recruiting key transcription factors like NRF1 and TFAM [43,44]. This process enables CAFs to maintain their mitochondrial network under stress. While more detailed studies in CRC CAFs are needed, the role of AMPK in promoting mitochondrial homeostasis and antioxidant defense—such as supporting NADPH and glutathione production—has been documented in other stromal contexts. This suggests a likely conserved function that helps CAFs of CRC manage metabolic stress.

### 3.5. PI3K/AKT–mTOR Signaling

The PI3K/AKT/mTOR axis is frequently activated within the tumor stroma, including CAFs in CRC [45]. In this context, AKT activation enhances glucose uptake, while mTORC1 promotes anabolic metabolism and protein synthesis. Notably, sustained mTOR signaling suppresses autophagy and may impair mitophagy, thereby contributing to the accumulation of dysfunctional mitochondria and elevated ROS levels [46]. Such metabolic alterations support CAF survival and reinforce their pro-tumorigenic secretory phenotype, notably the synthesis and secretion of trophic ligands as hepatocyte growth factor (HGF) and insulin-like growth factor 1 (IGF-1) [29]. These paracrine signals provide complementary inputs to cancer cells, promoting proliferation and therapeutic resistance. Molecular analyses further support the activation of this pathway within the CRC stromal compartment.

### 3.6. YAP/TAZ Mechanotransduction Pathway

The transcription co-activators YAP and TAZ serve as crucial sensors of the extracellular matrix’s physical properties. CAFs, which are typically found in regions of high matrix stiffness in CRC, demonstrate heightened YAP/TAZ activity [47,48,49]. This activation encourages a contractile and proliferative phenotype. Research in other contexts suggests that YAP/TAZ can directly affect mitochondrial dynamics, promote fission and increase ROS production. Additionally, YAP/TAZ activation in CAFs boosts the expression of glycolytic genes and lactate secretion, thereby enhancing the metabolic support provided to cancer cells. Although YAP/TAZ-mediated metabolic regulation has been extensively characterized in fibroblasts and CAFs from several tumor types, direct mechanistic evidence in CRC-associated fibroblasts remains limited. Nevertheless, the pronounced matrix stiffness and desmoplastic remodeling observed in CRC strongly suggest that YAP/TAZ-associated mechanotransduction may contribute to CAF metabolic adaptation in this context [50].

Moreover, recent studies further suggest that YAP/TAZ–TEAD signaling may contribute to adaptive therapeutic resistance in KRAS-mutant cancers. Genome-wide CRISPR screening analyses demonstrated that YAP/TAZ–TEAD pathway components represent synthetic-lethal vulnerabilities in the context of KRAS G12C inhibition, while KRAS inhibitor treatment itself may induce RHO/ROCK-dependent nuclear YAP activation [51]. Given the established roles of CAF-mediated matrix stiffness, mechanotransduction, and metabolic remodeling in regulating YAP/TAZ activity, these findings raise the possibility that stromal YAP/TAZ signaling may contribute to adaptive resistance through mitochondrial rewiring and metabolic crosstalk within the CRC microenvironment [52].

In conclusion, the mitochondrial phenotype of CAFs in colorectal cancer is shaped by a complex interplay of biochemical and biophysical signals from the TME. Some pathways, such as TGF-β/HIF-1α and ROS/NF-κB, are well-supported by evidence in CRC. Others, while not as directly validated in this specific context, are strongly suggested by the conserved biology of CAFs across various cancer types. Gaining a deeper understanding of how these pathways intersect and regulate stromal metabolism will be fundamental to the design of novel therapeutic interventions focused on modulating the TME and mitigating chemoresistance in colorectal cancer.

## 4. Functional Consequences of Mitochondrial Reprogramming in CRC-Associated Fibroblasts

For many years, aerobic glycolysis has long been considered a fundamental metabolic characteristic of cancer cells. However, recent research highlights the critical influence of the TME on cancer progression and the epithelial–mesenchymal transition. Within this intricate ecosystem, CAFs play a particularly vital role.

The metabolic and molecular interactions between tumor cells and fibroblasts significantly affect cancer cell proliferation, energy metabolism, metastatic potential, and overall disease progression. This relationship is exemplified by the “reverse Warburg effect” [29]. In this context, cancer cells secrete reactive oxygen species, particularly hydrogen peroxide, into the surrounding matrix, thereby inducing oxidative stress in adjacent fibroblasts. In a process of metabolic adaptation, fibroblasts transition toward aerobic glycolysis, thereby yielding energy-dense metabolites, including lactate and pyruvate [27].

**Figure 4:**
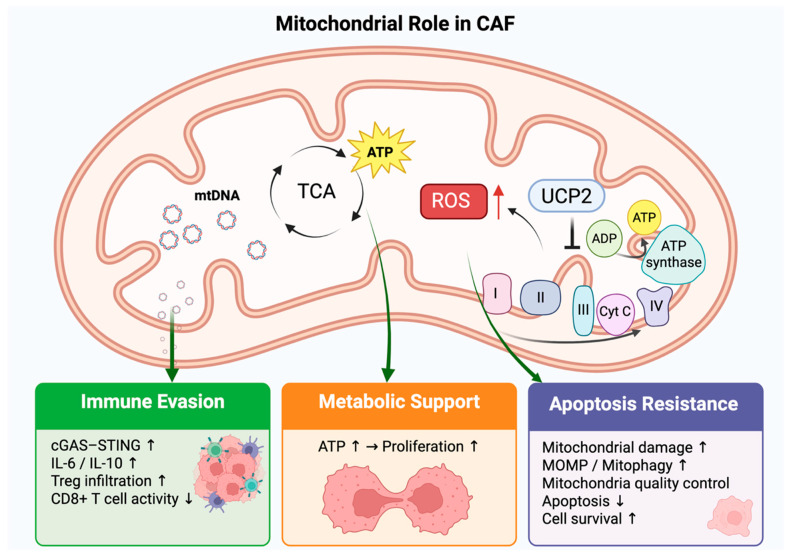
Functional consequences of mitochondrial remodeling in cancer-associated fibroblasts during colorectal cancer progression. Schematic illustration of how mitochondrial reprogramming in cancer-associated fibroblasts (CAFs) drives tumor progression and therapy resistance. Mitochondrial stress induces mtDNA release and activates the cGAS–STING pathway, promoting IL-6/IL-10 production and immunosuppressive Treg recruitment. Enhanced TCA cycle activity and ATP production provide metabolic support for tumor growth. Meanwhile, mitochondrial dysfunction triggers MOMP and mitophagy, but incomplete caspase activation results in apoptosis resistance. Upregulation of UCP2 further reduces mitochondrial ROS and supports redox adaptation. Collectively, these processes contribute to immune evasion, tumor growth, and resistance to cell death. Created in BioRender. Jeon, J. (2026) https://BioRender.com/fpewrh1 (accessd on 4 May 2026). Abbreviations: CAF, cancer-associated fibroblast; mtDNA, mitochondrial DNA; TCA, tricarboxylic acid cycle; ATP, adenosine triphosphate; ADP, adenosine diphosphate; ROS, reactive oxygen species; UCP2, uncoupling protein 2; Cyt c, cytochrome c; cGAS, cyclic GMP–AMP synthase; STING, stimulator of interferon genes; IL-6, interleukin-6; IL-10, interleukin-10; Treg, regulatory T cell; CD8+ T cell, CD8-positive T lymphocyte; MOMP, mitochondrial outer membrane permeabilization.

However, mitochondrial dysfunction in cancer-associated fibroblasts extends well beyond metabolic reprogramming. While the upstream signaling pathways driving mitochondrial remodeling have been discussed above, the functional consequences of this remodeling are equally critical. Increasing evidence suggests that mitochondria serve as a central signaling hub, and their dysfunction can trigger complex tumor-promoting mechanisms, including immunosuppression, chemotherapy resistance, and the regulation of cell death (Figure 4). Together, these processes may help establish a microenvironment that fosters tumor progression and contributes to treatment resistance [53].

The impairment of mitochondrial integrity triggers the translocation of mitochondrial DNA (mtDNA) into the cytoplasmic compartment. Once localized within the cytosol, mtDNA functions as a damage-associated molecular pattern (DAMP), where it is sensed by the enzyme cyclic GMP–AMP synthase (cGAS). This recognition event triggers the STING signaling cascade, which subsequently drives the expression of immunomodulatory cytokines, such as IL-6 and IL-10 [54,55,56]. These molecular signals promote the infiltration of regulatory T cells (Tregs) into the tumor site while concurrently blunting the effector functions of cytotoxic T lymphocytes, thereby promoting the establishment of an immunosuppressive TME that supports immune evasion.

In addition to immune signaling, mitochondria play a key role in maintaining cellular redox balance and survival. For instance, increased expression of mitochondrial uncoupling protein 2 (UCP2) disrupts the proton gradient across the mitochondrial membrane, which decreases mitochondrial ROS generation and reduces the efficiency of oxidative phosphorylation (OXPHOS) [57,58]. Notably, although CAFs are initially exposed to elevated oxidative stress induced by tumor-derived ROS, they progressively adapt by enhancing antioxidant systems to buffer excessive ROS levels [59]. At the same time, enhanced synthesis of glutathione (GSH) strengthens cellular antioxidant capacity. Collectively, these adaptations help cells cope with therapy-induced oxidative stress and limit apoptosis [60]. Additionally, alterations in mitochondrial outer membrane permeability regulate the efflux of pro-apoptotic proteins, particularly cytochrome c (CytC), into the cytoplasmic space, whereas persistent survival signaling can prevent full activation of the caspase cascade, ultimately favoring an anti-apoptotic cellular state [61,62].

This characteristic enables CAFs to persist within the harsh tumor microenvironment and continuously support cancer development.

Through these mitochondria-mediated mechanisms, CAFs in colorectal cancer contribute to a microenvironment characterized by immunosuppression, therapeutic resistance, and enhanced tumor growth, underscoring their potential as promising therapeutic targets.

## 5. Clinical Implications of CAF-Mediated Mitochondrial Reprogramming in CRC Progression

Based on the aforementioned key mechanisms of CAF mitochondrial metabolism, its role in mediating chemotherapy resistance in CRC has become increasingly clear. This metabolic reprogramming not only alters the functions of CAFs themselves, but also directly or indirectly helps tumor cells evade the killing effect of chemotherapy drugs through metabolic exchange, signal transduction, and other means (Table 1).

### 5.1. Chemotherapy Resistance

#### 5.1.1. Mitochondrial Metabolic Reprogramming in CAFs

CAFs undergo profound metabolic reprogramming compared with their normal counterparts [84,85]. In CAFs, there is a widely supported model of metabolic reprogramming, namely the “Reverse Warburg effect”, which suggests that CAFs mainly undergo aerobic glycolysis (high glucose uptake and lactate production), meaning that even under aerobic conditions, the metabolism is reprogrammed to aerobic glycolysis. This process mirrors the Warburg effect described in cancer cells, except that it occurs in stromal cells. Their basal oxygen consumption is reduced, and mitochondrial function is impaired [86]. And studies have shown that this oxidative stress in the CAFs promotes the metabolism and mutagenic activity of tumor cells [87]. Specifically, stromal fibroblasts generate reactive oxygen species (ROS) and transition to aerobic glycolysis, facilitating the accumulation of high-energy metabolites, including L-lactate and ketone bodies [88]. These metabolic substrates are readily assimilated by neoplastic cells to fuel mitochondrial oxidative phosphorylation, thereby sustaining their bioenergetic requirements, and can be taken up by CRC cells as alternative carbon sources. In addition, mitochondrial enzymes like isocitrate dehydrogenase 2 (IDH2) and malic enzyme 2 (ME2) are often upregulated in CAFs, facilitating NADPH production and maintaining redox balance [89]. Overall, the reprogrammed mitochondrial metabolism of CAFs creates a supportive metabolic area that enhances tumor growth and survival under stress conditions such as chemotherapy.

#### 5.1.2. Redox Homeostasis and Antioxidant Defense

CAFs rely heavily on mitochondrial generation of reactive oxygen species (ROS), and proper redox balance is required to sustain CAF function and protect tumor cells [90]. To counteract oxidative stress, CAFs upregulate multiple antioxidant pathways, including the NRF2-mediated transcriptional program and the glutathione (GSH) system [91]. Enhanced NADPH production through mitochondrial enzymes sustains GSH recycling and preserves a reduced intracellular environment. This robust antioxidant capacity allows CAFs to survive under high levels of oxidative stress and, importantly, to buffer therapy-induced ROS in the tumor microenvironment. The mitochondrial uncoupling protein UCP2 may also participate in this process by reducing ROS generation through mild uncoupling, although direct evidence in colorectal CAFs is still limited [92]. In addition, CAFs can remove damaged mitochondria through mitophagy, further reducing oxidative stress. Collectively, these mechanisms establish a strong antioxidant barrier that not only protects CAFs but also indirectly shields nearby colorectal cancer cells from chemotherapy-induced oxidative damage.

#### 5.1.3. CAF-Derived Metabolic and Signaling Support in Chemoresistance

Beyond their intrinsic metabolic reprogramming, CAFs actively communicate with CRC cells through both metabolic and paracrine interactions, shaping a microenvironment that promotes chemoresistance [29,93,94]. CAFs facilitate the secretion of various metabolites, including lactate, pyruvate, and glutamine, which are subsequently internalized by malignant cells and shuttled into the mitochondrial respiratory chain to support bioenergetic requirements [95]. This metabolic synergy not only furnishes supplementary energy to sustain viability under physiological stress but also facilitates the regulation of redox homeostasis and anabolic flux, thereby augmenting the chemoresistance of neoplastic cells [96]. Beyond the provision of metabolic substrates, CAFs discharge a diverse repertoire of cytokines and growth factors—most notably IL-6, IL-8, IGF-1, and HGF—which orchestrate the activation of critical survival-associated signaling networks within the tumor cell population [97]. These pathways, notably PI3K/AKT, MAPK/ERK, and STAT3, enhance anti-apoptotic mechanisms, stimulate DNA repair, and promote stem-like phenotypes, all of which are closely linked to drug resistance [98,99]. Distinctively, CAF-derived Wnt ligands are capable of stimulating the canonical Wnt/β-catenin signaling axis within CRC cells. This activation facilitates the nuclear translocation and subsequent accumulation of β-catenin [100,101], where it recruits TCF/LEF transcription factors to orchestrate the expression of genetic programs governing cellular stemness, the epithelial–mesenchymal transition (EMT), and pro-survival mechanisms [102]. This transcriptional reprogramming enhances not only resistance to conventional chemotherapeutics like oxaliplatin and 5-fluorouracil but also adaptive responses to targeted therapies.

Moreover, CAFs can modulate β-catenin activity through indirect mechanisms. For instance, CAF-derived IL-6 and IGF-1 signaling can lead to β-catenin phosphorylation and nuclear translocation via AKT-mediated pathways [63,64]. Exosomal transfer of non-coding RNAs from CAFs has also been shown to stabilize β-catenin in cancer cells, further reinforcing its transcriptional program. Collectively, this metabolic and signaling circuits governed by CAFs foster the development of a resilient microenvironmental niche, effectively shielding malignant cells from chemotherapy-induced damage, oxidative stress and further promotes long-term adaptation, contributing significantly to therapy failure in CRC.

#### 5.1.4. Emerging Mitochondrial Crosstalk Mechanisms

Communication between CAFs and cancer cells mediated by mitochondria is not limited to conventional metabolic exchange, as suggested by recent studies. CAFs can transfer mitochondrial components, mitochondrial DNA, or even intact mitochondria to neighboring tumor cells through tunneling nanotubes or extracellular vesicles [103]. These transferred organelles can restore mitochondrial respiration and increase ATP production in stressed cancer cells, thus promoting survival under chemotherapy [104]. Furthermore, mitochondrial stress in CAFs may promote the release of mitokines and reactive metabolites that modify tumor cell behavior at a distance [105]. CAFs may also influence local drug bioavailability through enhanced autophagic flux or upregulation of drug efflux transporters, indirectly reducing the effective concentration of chemotherapeutic agents [106]. Emerging evidence further suggests that CAF-associated metabolic adaptation may also contribute to irinotecan resistance in CRC. Metabolic stress and glutamine-dependent mitochondrial remodeling have been implicated in sustaining tumor cell survival and stemness under SN38 treatment, while mitochondrial fragmentation-associated metabolic plasticity may further support adaptive resistance mechanisms [65,66,67]. Although these findings are relatively new, they highlight the dynamic and bidirectional mitochondrial communication within the TME, suggesting that targeting CAF mitochondrial function could be a promising strategy to overcome chemoresistance in CRC.

### 5.2. Immunotherapy Resistance

While CAFs contribute significantly to chemotherapy resistance through metabolic reprogramming and paracrine signaling, their influence extends beyond conventional cytotoxic therapies. Increasing evidence suggests that CAFs also play a key role in shaping the tumor immune microenvironment, thereby limiting the efficacy of immunotherapies [107]. Similar mechanisms that allow CAFs to protect tumor cells from chemotherapeutic stress—such as secretion of growth factors, cytokines, and metabolic intermediates—can also suppress anti-tumor immune responses [108,109]. In addition, recent studies indicate that CAFs can modulate innate immune sensing pathways, including the mitochondrial DNA (mtDNA)/cGAS–STING axis, adding another layer of complexity to immune evasion in CRC [56,110]. CAFs modulate the immune microenvironment through both paracrine and metabolic interactions, they orchestrate the release of a broad spectrum of immunosuppressive factors—most notably TGF-β, IL-6, and CXCL12—which work in concert to diminish the recruitment and diminish the anti-tumor potency of cytotoxic T cells. Concurrently, these signaling molecules drive the proliferation of regulatory T cell populations and orchestrate the infiltration of myeloid-derived suppressor cells (MDSCs) into the tumor stroma [111,112]. Through these mechanisms, CAFs establish a local immunosuppressive niche that protects tumor cells from immune-mediated killing. Beyond soluble factors, CAF mitochondria can actively influence immune responses. Stress or metabolic reprogramming in CAFs can trigger the release of mtDNA, either directly into the extracellular space or packaged within exosomes [113]. Upon its release, mtDNA acts as a ligand for the cytosolic sensor cyclic GMP-AMP synthase (cGAS) within the tumor or adjacent immune cells; this recognition triggers the STING signaling axis, ultimately catalyzing the expression of type I interferons and a broad array of inflammatory cytokines [54,114], and chronic activation of inflammatory pathways such as cGAS–STING and NF-κB have been associated with T-cell dysfunction, immunosuppressive microenvironment remodeling, and reduced responsiveness to anti-PD-1/PD-L1 therapies [68,69,70,71]. In addition, mitochondrial metabolic remodeling and ECM-associated stromal activation may further impair anti-tumor immune infiltration and contribute to resistance to CTLA-4-targeted immunotherapy through TGF-β-dependent immunosuppressive signaling and persistent stromal remodeling [72,73,74]. Although this pathway is typically part of the innate immune defense, sustained or dysregulated activation can paradoxically promote chronic inflammation and recruit immunosuppressive cell populations, ultimately reducing the effectiveness of immune checkpoint therapies.

Beyond mtDNA-mediated signaling, mitochondrial metabolic activities within CAFs exert a profound influence on the tumor landscape by modulating the local nutrient pool, preserving redox homeostasis, and generating metabolic byproducts that tune immune cell behavior [40,115,116]. Collectively, these integrated pathways establish a permissive microenvironmental niche that facilitates immune evasion and confers resistance to immunotherapeutic interventions. Understanding how CAFs utilize mitochondrial signals, particularly the mtDNA–cGAS–STING pathway, provides new insights into immune evasion and offers potential strategies to enhance the effectiveness of immunotherapies in colorectal cancer. In conclusion, translational translation of CAF-targeted metabolic inhibitors faces the dual challenge of specificity and systemic toxicity. In future, targeting CAF-associated metabolic adaptations, particularly those involving mitochondrial dynamics and metabolite exchange, may represent a promising approach to alleviate immunosuppressive remodeling of the microenvironment and improve responses to immunotherapy in CRC.

### 5.3. Additional Therapeutic Implications of CAF Mitochondrial Remodeling

Accumulating evidence suggests that mitochondrial metabolic adaptation within the tumor microenvironment may also contribute to resistance to targeted therapies in CRC. CAF-mediated lactate exchange and mitochondrial stress signaling have been associated with compensatory activation of survival pathways such as STAT3, thereby reducing the efficacy of EGFR-targeted therapies including cetuximab [75]. In addition, adaptive mitochondrial rewiring and glutamine-dependent metabolic flexibility may support tumor cell survival during BRAF/MEK inhibitor treatment and promote metabolic adaptation under therapeutic pressure [76].

**Figure 5:**
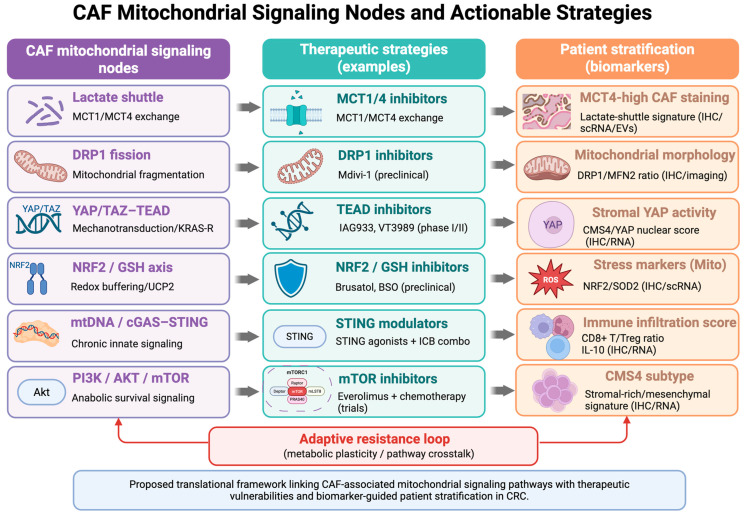
Translational roadmap for CAF mitochondrial targeting in CRC. Each row represents one CAF mitochondrial signalling node linked to a candidate therapeutic strategy and a corresponding patient stratification biomarker. The bottom row (shaded red) highlights the adaptive resistance loop: metabolic plasticity and crosstalk between nodes may undermine single-agent targeting, supporting the rationale for combination strategies and longitudinal biomarker monitoring. Created in BioRender. Jeon, J. (2026) https://BioRender.com/tepk0qz (accessd on 4 May 2026). Abbreviations: KRAS-R = KRAS inhibitor resistance; CMS4 = consensus molecular subtype 4 (stromal/mesenchymal); ICB = immune checkpoint blockade; IHC = immunohistochemistry; scRNA = single-cell RNA sequencing; EVs = extracellular vesicles. DRP1, Dynamin-Related Protein 1; YAP/TAZ–TEAD, Yes-Associated Protein/Transcriptional Co-Activator with PDZ-Binding Motif–TEA Domain Transcription Factors; NRF2/GSH, Nuclear Factor Erythroid 2–Related Factor 2/Glutathione; mtDNA, mitochondrial DNA; cGAS–STING, cyclic GMP–AMP synthase–stimulator of interferon genes; PI3K/AKT/mTOR, phosphoinositide 3-kinase/protein kinase B/mechanistic target of rapamycin; MCT1/4, monocarboxylate transporter 1 and monocarboxylate transporter 4. Evidence levels for each node are detailed in Table 2.

Mitochondrial antioxidant defenses and hypoxia-associated metabolic remodeling may further contribute to resistance to radiotherapy and anti-angiogenic therapy. Enhanced ROS buffering, mitochondrial stress responses, and mtDNA-associated signaling have been linked to radioresistance and tumor cell survival following irradiation [77,78,79]. Under hypoxic conditions, CAF-associated metabolic adaptation can also promote lactate production, VEGF signaling, and extracellular matrix remodeling, thereby supporting resistance to anti-angiogenic therapies such as bevacizumab [80,81,82,83]. Collectively, these findings suggest that multiple CAF-associated mitochondrial signaling pathways may represent actionable therapeutic targets and potential biomarkers for patient stratification in CRC (Figure 5).

**Table 2:** Evidence levels for key CAF mitochondrial mechanisms.

Pathway/ Mechanism	Evidence Level	Supporting Basis	Refs
TGF-β/HIF-1α-driven metabolic reprogramming in CRC CAFs Section 3.1	Direct CRC CAF evidence	TGF-β and HIF-1α contributions to metabolic reprogramming validated in CRC tumor and stromal compartments.	[36,37]
ROS/NF-κB → cytokine secretion (IL-6, IL-8, CXCL12) Section 3.2	Direct CRC CAF evidence	Documented in CRC CAF models; consistent with stromal oxidative stress studies.	[37,38,39,40]
Reverse Warburg effect/lactate shuttle (MCT1/MCT4) Section 2.2 and Section 5.1	Direct CRC CAF evidence	Demonstrated in CRC stromal–tumor metabolic exchange studies	[26,27,96]
DRP1-mediated mitochondrial fission in CRC cells Section 2.1	CRC tumor cell evidence	Well established in CRC tumor cells; evidence in CRC CAFs specifically remains limited.	[12,15,18]
PI3K/AKT/mTOR activation in CRC stroma Section 3.5	CRC tumor cell evidence	Pathway activation documented in CRC; direct CAF mitochondrial link inferred from tumor cell studies.	[45,46]
NRF2/GSH antioxidant defense in CAF chemoresistance Section 5.1.2	CRC tumor cell evidence	NRF2 and GSH roles established in CRC; direct UCP2 evidence in CRC CAFs limited.	[64,92]
YAP/TAZ mechanotransduction → metabolic regulation Section 3.6	CAF evidence (other cancers)	Extensively characterised in CAFs from breast and pancreatic cancers; inferred in CRC based on desmoplastic microenvironment.	[47,48,49,50,51,52]
AMPK/PGC-1α → mitochondrial biogenesis in CAFs Section 3.4	CAF evidence (other cancers)	Conserved stromal energy-sensing program; CRC CAF-specific validation lacking.	[43,44]
Mitochondrial transfer via tunneling nanotubes/EVs Section 5.1.4	CAF evidence (other cancers)	Demonstrated in breast CAF models; mechanistic relevance in CRC is plausible but not directly tested.	[103,104]
mtDNA/cGAS–STING in CRC CAF immunosuppression Section 3.3	Hypothesis/future direction	Pathway established in other stromal systems; direct evidence in CRC-associated fibroblasts currently lacking; requires experimental validation.	[54,55,56]
CAF-derived metabolic modelling/spatial metabolic flux Section 5.4	Hypothesis/future direction	Computational and spatial single-cell approaches proposed; not yet applied to CRC CAF mitochondrial phenotypes.	[20,28]

Evidence levels: Direct CRC CAF evidence = validated in CRC-associated fibroblasts; CRC tumor cell evidence = demonstrated in CRC cells, extrapolated to CAFs; CAF evidence (other cancers) = validated in other tumor CAFs, inferred in CRC; Hypothesis/future direction = mechanistically plausible, currently lacking direct CRC CAF validation. Abbreviations: CAF, cancer-associated fibroblast; CRC, colorectal cancer; DRP1, dynamin-related protein 1; MCT, monocarboxylate transporter; NRF2, nuclear factor erythroid 2-related factor 2; GSH, glutathione; UCP2, uncoupling protein 2; YAP/TAZ, Yes-associated protein/transcriptional coactivator with PDZ-binding motif; AMPK, AMP-activated protein kinase; PGC-1α, peroxisome proliferator-activated receptor gamma coactivator 1-alpha; PI3K, phosphoinositide 3-kinase; mtDNA, mitochondrial DNA; cGAS–STING, cyclic GMP–AMP synthase–stimulator of interferon genes; EVs, extracellular vesicles; IHC, immunohistochemistry; scRNA, single-cell RNA sequencing. Taken together, these findings suggest that multiple CAF-associated mitochondrial signaling pathways may serve as clinically actionable therapeutic targets and potential biomarkers for patient stratification in CRC.

### 5.4. Limitations and Future Perspectives

Despite the growing interest in CAF mitochondrial remodeling, several limitations remain in the current field. Many mechanistic insights are derived from studies conducted in other tumor types or generalized stromal models, while direct CRC-specific evidence remains comparatively limited for certain signaling pathways. In addition, the marked heterogeneity and plasticity of CAF populations continue to complicate the interpretation of metabolic phenotypes and therapeutic vulnerabilities. Future investigations integrating spatial and single-cell metabolic approaches will be essential to better define subtype-specific mitochondrial adaptations within the CRC microenvironment.

Recent advances in metabolic modeling and single-cell computational analyses have further highlighted the potential contribution of CAF-driven metabolic heterogeneity to CRC progression [20]. Predictive metabolic network modeling may help identify subtype-specific nutrient dependencies, metabolic coupling patterns, and adaptive mitochondrial programs within the tumor microenvironment, thereby providing additional opportunities for precision stromal-targeted therapies. In addition to targeting canonical oncogenic pathways, increasing attention has been directed toward therapeutic strategies aimed at altered energy metabolism in CRC, including inhibition of glycolysis, lactate transport, glutamine utilization, and mitochondrial oxidative phosphorylation [117]. Combining metabolic interventions with conventional chemotherapy or immunotherapy may provide opportunities to overcome stromal-mediated therapeutic resistance.

## 6. Conclusions

CRC progression is profoundly shaped by the complex metabolic and signaling interactions between malignant cells and stromal components, particularly CAFs. Accumulating evidence suggests that mitochondrial reprogramming within CAFs is not merely a passive response to tumor-induced stress but an active contributor to tumor progression, metabolic symbiosis, therapeutic resistance, and immune modulation within the CRC tumor microenvironment. CAFs construct a metabolically supportive microenvironment by fundamentally altering mitochondrial dynamics, which enhances glycolytic activity and strengthens antioxidant defenses, thereby shielding cancer cells against chemotherapy-induced oxidative stress. Beyond cell-intrinsic effects, mitochondrial signaling in CAFs is a critical determinant of an immunosuppressive TME. Mitochondrial signaling in CAFs may also contribute to chronic inflammation and immune suppression within the CRC tumor microenvironment.

In sum, these findings establish CAF mitochondria as a pivotal signaling hub that integrates metabolic, inflammatory, and survival-promoting inputs within the CRC TME. Consequently, selectively disrupting these spectra of CAF-driven tumor-promoting functions via mitochondrial targeting holds substantial therapeutic potential to overcome multi-drug resistance. Future endeavors must transition from static, simplified models to a more dynamic understanding of CAF functional diversity. Leveraging in situ, high-resolution technologies, such as spatial multi-omics and single-cell metabolic profiling, will be critical to dissect the contextual coordination between mitochondrial alterations, signaling networks, and metabolic flux under varying therapeutic conditions. From a translational perspective, while targeting stromal metabolism remains an attractive avenue, the primary challenge lies in enhancing precision to minimize systemic toxicity. A comprehensive framework of CAF metabolic heterogeneity and mitochondrial regulation is ultimately essential to realize effective and clinically relevant interventions for CRC.

## 7. Literature Search Strategy

Literature for this narrative review was primarily retrieved from PubMed, Web of Science, and Google Scholar databases using combinations of keywords including “colorectal cancer”, “cancer-associated fibroblasts”, “mitochondrial reprogramming”, “metabolic remodeling”, and “tumor microenvironment”. Preference was given to recent mechanistic and translational studies relevant to CAF-associated mitochondrial remodeling, metabolic adaptation, and therapeutic resistance in CRC.

## Figures and Tables

**Table 1:** Metabolic resistance to cancer therapies driven by CAF mitochondrial reprogramming in CRC.

Resistance Type	Therapies/Drugs	CAF Mitochondrial Mechanisms	Affected Cell Type	Metabolites Involved	Upstream Trigger	Downstream Pathway	Biological Effect/Significance
Chemoresistance	Oxaliplatin	Enhanced mitochondrial NADPH/GSH axis detoxifies ROS; UCP2 reduces mito-ROS; lactate/pyruvate shuttle supports tumor OXPHOS	Tumor cells, CAFs	GSH, NADPH, lactate, pyruvate	Chemotherapy-induced oxidative stress; TGF-β	NRF2, AKT, Wnt-related ROS adaptation	Reduced DNA damage; sustained tumor survival under oxidative stress [63,64]
	5-Fluorouracil (5-FU)	Mitochondrial folate metabolism provides formate for cytosolic nucleotide synthesis, while mitochondrial ROS induces IL-6 secretion; DRP1-driven mitochondrial fission enhances CAF survival	Tumor cells, CAFs	Formate, GSH, mitochondrial folate intermediates	TGF-β; inflammatory cytokines	STAT3, MAPK, p38	Enhanced proliferation, survival signaling, accelerated DNA repair [63,64]
	Irinotecan (SN38)	CAF mitochondrial OXPHOS supports metabolic plasticity; glutamine transfer fuels CRC OXPHOS	Tumor cells	Glutamine, α-KG	Metabolic stress	mTORC1, MYC metabolic program	Increased ATP production; suppressed apoptosis [65,66,67]
Immunotherapy Resistance	Anti-PD-1/Anti-PD-L1	mtDNA release → chronic, non-productive cGAS–STING activation; lactate impairs T cell function and promotes an immunosuppressive niche; mito-ROS → IL-6 → PD-L1 upregulation	CD8^+^ T cells, Tregs, tumor cells	mtDNA, lactate, ROS	Hypoxia; mitochondrial stress; inflammatory cues	cGAS–STING, STAT3, NF-κB	T cell exhaustion; immunosuppressive TME; PD-L1 elevation [68,69,70,71]
	Anti-CTLA4	Mito-driven proline metabolism → ECM stiffening; TGF-β sustained by mitochondrial activity	CD4^+^/CD8^+^ T cells	Proline, hydroxyproline	Hypoxia, ECM stress	TGF-β/SMAD, FAK	T cell exclusion; reduced immune infiltration [72,73,74]
Targeted Therapy Resistance	Anti-EGFR (Cetuximab)	Mitochondria-dependent lactate/ketone export bypasses EGFR dependency; mito-stress induces HGF	Tumor cells	Lactate, ketone bodies	Drug-induced signaling blockage	MAPK, STAT3	Maintains growth signaling despite EGFR inhibition [75]
	BRAF/MEK inhibitors	Glutamine transfer and OXPHOS rebound driven by CAF mitochondria	Tumor cells	Glutamine, malate, citrate	Adaptive metabolic stress	OXPHOS-related signaling; ERK rebound	Rapid adaptive resistance; metabolic rewiring [76]
Radiotherapy Resistance	Ionizing radiation	Mitochondrial antioxidant defense (SOD2, GSH) neutralizes radiation-induced ROS; mtDNA signaling promotes stemness	Tumor cells, immune cells	ROS, GSH, mtDNA	Radiation-induced mitochondrial injury	STING, NRF2	Reduced apoptosis; increased tumor survival after irradiation [77,78,79]
Anti-angiogenic Resistance	Bevacizumab	Hypoxia → CAF mitochondrial activation → lactate/VEGF production; mito-support for ECM remodeling	Endothelial cells, tumor cells	Lactate, VEGF, succinate	Hypoxia	HIF-1α	Angiogenesis rebound; hypoxia-driven tumor progression [80,81,82,83]

The mechanistic pathways summarized in this table are based on findings from CRC studies and mechanistically related tumor models cited throughout the manuscript. Abbreviations: Colorectal cancer (CRC); cancer-associated fibroblast (CAF); nicotinamide adenine dinucleotide phosphate (NADPH); glutathione (GSH); reactive oxygen species (ROS); uncoupling protein 2 (UCP2); oxidative phosphorylation (OXPHOS); transforming growth factor-β (TGF-β); nuclear factor erythroid 2-related factor 2 (NRF2); AKT serine/threonine kinase (AKT); interleukin-6 (IL-6); dynamin-related protein 1 (DRP1); signal transducer and activator of transcription 3 (STAT3); mitogen-activated protein kinase (MAPK); alpha-ketoglutarate (α-KG); mechanistic target of rapamycin complex 1 (mTORC1); MYC proto-oncogene, bHLH transcription factor (MYC); programmed cell death protein 1 (PD-1); programmed cell death-ligand 1 (PD-L1); cluster of differentiation 4-positive T cells (CD4+); cluster of differentiation 8-positive T cells (CD8+); mitochondrial DNA (mtDNA); cyclic GMP-AMP synthase–stimulator of interferon genes (cGAS–STING); nuclear factor kappa B (NF-κB); tumor microenvironment (TME); extracellular matrix (ECM); SMAD family member proteins (SMAD); focal adhesion kinase (FAK); epidermal growth factor receptor (EGFR); hepatocyte growth factor (HGF); B-Raf proto-oncogene serine/threonine kinase/mitogen-activated protein kinase kinase (BRAF/MEK); superoxide dismutase 2 (SOD2); vascular endothelial growth factor (VEGF); hypoxia-inducible factor 1 alpha (HIF-1α).

## Data Availability

No new datasets were generated or analyzed during the current study. Data sharing is not applicable to this article.
